# Retroperitoneal Extragonadal Nonseminomatous Germ Cell Tumor with Synchronous Orbital Metastasis

**DOI:** 10.1155/2009/419059

**Published:** 2009-01-29

**Authors:** Ali Fuat Atmaca, Serkan Altınova, Abdullah Erdem Canda, M. Fuat Ozcan, Suleyman Alıcı, Leyla Memıs, M. Derya Balbay

**Affiliations:** ^1^1st Urology Department, Ankara Ataturk Training and Research Hospital, Ankara, Turkey; ^2^Ufuk Universitesi, Cad. no: 22/26 Çukurambar, Çankaya, 06520 Ankara, Turkey; ^3^Medical Oncology Department, Ankara Ataturk Training and Research Hospital, Ankara, Turkey; ^4^Department of Pathology, School of Medicine, Gazi University, 06500 Ankara, Turkey

## Abstract

A huge retroperitoneal tumor with a right orbital mass was detected and proved to be an extragonadal nonseminomatous germ cell tumor on biopsy. BEP chemotherapy caused some regression in orbital mass however no change in retroperitoneal tumor size as well as serum tumor marker levels occurred. Herein, we present a rarely seen entity of extragonadal retroperitoneal nonseminomatous germ cell tumor with synchronous orbital metastases and discuss its diagnosis and management.

## 1. Introduction

Extragonadal germ cell tumors (EGGCT) account for approximately 2–5% of all germ cell tumors. They are mostly seen in the mediastinum and retroperitoneum. However, it can also be seen in less frequent locations such as sacrococygeal region, pineal region, prostate, orbita, liver, vagina, and gastrointestinal tract [1, 2].

These lesions may grow up to extensive dimensions without any significant symptoms. The diagnosis is usually made in the third decade. Advanced local disease and distant metastases might exist at the time of diagnosis in the majority of patients [3].

## 2. Case Report

A 31-year-old man complaining of bilateral lumber pain, fatigue was diagnosed with right hydronephrosis on ultrasonography (US), and a double J stent was placed into the right ureter at another hospital a month ago. He then was referred to our institution for further evaluation. His past medical history revealed bilateral nephrolithotomy in 1998 and right nephrolithotomy in 1999. Physical examination of urogenital system was within normal limits including the scrotal examination. On admission, there was proptosis (eccentric) present displacing his right eye infero-nasally. His blood hemoglobulin and leukocyte counts were 7.81 g/dL and 21 500, respectively. Blood chemistry was within normal limits except elevated urea (49 mg/dL) and creatinine (2.1 mg/dL) levels. Serum tumor marker studies revealed an increased beta-human chorionic gonadotropin (*β* HCG) level (37.8 mIU/mL) and an increased lactate dehydrogenase (LDH) level (800 IU/L). Serum alpha fetoprotein (AFP) level was within normal limits.

Abdominal doppler US has shown multiple bilateral renal stones, grade 2 ectasia in the left kidney and multiple irregular iso-hyperechoic hypervascular solid lesions in the right kidney. Additionally, scrotal US examination revealed no abnormalities.

**Figure 1:**
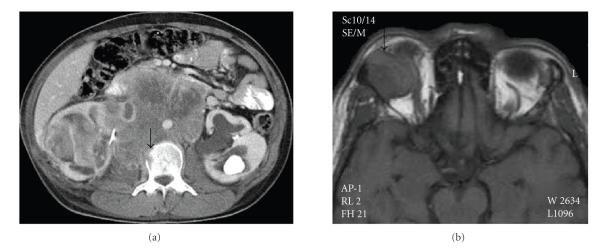
(a) CT of the retroperitoneal mass. Note the vertebral body invasion (arrow). CT: Computerized tomography. (b) T1-weighted MRI scans of the right orbital mass (arrow). MRI: Magnetic resonance imaging.

On computerized tomography scans, multiple heavily contrast-stained conglomaralated necrotic lymphadenopathies undifferentiated from right kidney in paraaortic and paracaval regions and destruction of L2 vertebral body were observed (Figure 1(a)). On magnetic resonance imaging, a contrast stained mass of 23 × 20 × 18 mm in size displacing superior and lateral orbital rectus muscles and also infiltrating into the frontal bone was seen in the right orbita (Figure 1(b)).

**Figure 2:**
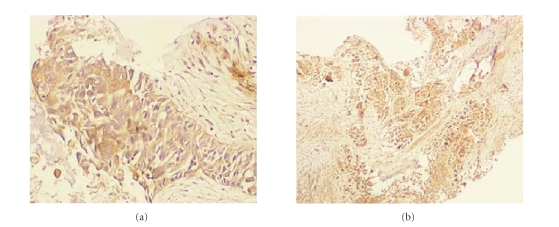
(a) Microscopic appearance of tumor cells showing diffuse and strong positive immunohistochemical staining for alpha fetoprotein. (b) Microscopic appearance of tumor cells showing diffuse and strong positive immunohistochemical staining for beta human chorionic gonadotropin.

Fine needle aspiration cytology from retroperitoneal mass demonstrated a carcinoma associated with germ cell tumor with positive staining for both AFP and *β*-HCG on immunohistochemical evaluation (Figures 2(a) and 2(b)).

The diagnosis was made as primary nonseminomatous extragonadal (retroperitoneal) germ cell tumor (EGGCT) with orbital metastasis. Chemotherapy including cisplatin, etoposide, and bleomycine (BEP) was administered. After completion of first course chemotherapy, symptoms subsided with a significant reduction in proptosis. However, serum tumor marker levels did not change. Afterwards, a very rapid progression of the disease has occurred following initial regression of his symptoms. Dimensions of orbital lesion increased significantly with concomitant increase in serum tumor marker levels including AFP and *β*-HCG. Unfortunately, the patient died after the second course of chemotherapy.

## 3. Discussion

EGGCTs are rarely seen tumors with specific biological and clinical characteristics. Symptoms depend on the location of the tumor such as presence of a palpable mass, abdominal or back pain, dysphagia, and edema in the limbs when the tumor is located in the retroperitoneum. Constitutional symptoms such as fever and weight loss might accompany the disease. The diagnosis is made histopathologically when seminomatous and nonseminomatous elements of the tumor are seen on biopsy. Tumor markers are also expected to increase if nonseminomatous elements are present [1, 2].

In our patient, main symptoms were abdominal and low back pain. Immunohistochemical evaluation of the biopsy for AFP and HCG staining were positive therefore, the patient was diagnosed as having extragonadal nonseminomatous germ cell tumor.

Metastases to other tissues depend on the localization and histological type of the primary tumor [4]. Lung metastasis rate has been reported to be 27% in mediastinal nonseminomatous tumors whereas it has been reported to be 49% in retroperitoneal tumors [4]. Additionally, abdominal (34%), liver (25%), and cervical lymph node (18%) metastases might also be present [4].

Scrotal US could easily differentiate a retroperitoneal EGGCT from primary testicular tumor metastases without routinely performing testicular biopsy for differential diagnosis [2].

The prognosis is excellent in cases with seminomatous histology regardless of the localization of the EGGCT either in mediastinum or in retroperitoneum. However, prognosis of nonseminomatous EGGCT is worse than that of the seminomatous variant. Five-year-survival rates have been reported to be 45% for mediastinal and 62% for retroperitoneal nonseminomatous tumors [2, 4]. Unfortunately, the majority of patients (80%) have nonseminomatous EGGCT thus a poor prognosis which is independent of the primary location of the nonseminomatous EGGCT [2, 4].

Management of patients with EGGCT is accomplished according to the prognostic classification of International Germ Cell Cancer Collaboration Group [2]. Standard cisplatinum-based chemotherapy plus additional secondary surgery in half of the patients is the recommended treatment strategy. Salvage chemotherapy including high-dose chemotherapy does not have a significant impact on long-term survival. Regarding seminomatous tumors, 3 courses of BEP chemotherapy administration have been suggested for patients with good prognosis, and 4 courses of BEP chemotherapy have been recommended for patients with moderate prognosis. In retroperitoneal nonseminatous tumors, 3 and 4 courses of BEP chemotherapy regimen should be administered to patients with good or moderate and poor prognostic criteria, respectively. In patients with mediastinal EGGCT, 4 courses of BEP chemotherapy are suggested [2].

Secondary surgery is an integral part of the treatment strategy in patients with EGGCT and is mandatory in cases with residual mass, which is true for about 50% of the patient population [2].

We intended to treat our patient with 4 courses of BEP chemotherapy; however, we were able to administer only two courses. Unfortunately, we lost the patient despite partial response with some regression in the diameter of the orbital mass without any change in serum tumor marker levels.

In our case, a right orbital mass accompanying the retroperitoneal tumor which we think was metastasis rather than another primary focus regressed after the administration of first course of BEP chemotherapy. Because the volume of the orbital tumor mass was smaller than the volume of the retroperitoneal tumor, we think that orbital tumor regressed to some degree while the retroperitoneal mass remained unchanged without any change in serum tumor marker levels.
